# Supplementary material for: The Systems Biology Research Tool: evolvable open-source software
Source: BMC Syst Biol. 2008 Jun 29;2:55. doi: 10.1186/1752-0509-2-55 (PMC2446383; doi:10.1186/1752-0509-2-55)
Supplement: Additional file 1 — SBRT Archive. An archive of the current version of the Systems Biology Research Tool. [file 1752-0509-2-55-S1.zip › sbrt-1.4.0/doc/developers_guide/api/sbrt/kernel/fba/proc/FbaSolverProcess.html]

FbaSolverProcess


|  |  |  |
| --- | --- | --- |
| **Overview** \| **Package** \| **Class** \| **Use** \| **Tree** \| **Deprecated** \| **Index** \| **Help** | |  |
| **PREV CLASS**   **NEXT CLASS** | **FRAMES**    **NO FRAMES**     **All Classes** |  |
| SUMMARY: NESTED \| FIELD \| CONSTR \| METHOD | DETAIL: FIELD \| CONSTR \| METHOD |  |


---


## sbrt.kernel.fba.proc Class FbaSolverProcess

```
java.lang.Object
  sbrt.kernel.fba.proc.FbaProcess<LinearSystemSolution<java.lang.String>>
      sbrt.kernel.fba.proc.FbaSolverProcess
```

**All Implemented Interfaces:**: KernelProcess<LinearSystemSolution<java.lang.String>>

---

``` public final class FbaSolverProcess extends FbaProcess<LinearSystemSolution<java.lang.String>> ```

This class is used to solve Sv = 0.

**Author:**
:   This class was written and documented by Jeremiah Wright while in the
    Wagner lab.

**See Also:**: `FbaSolver`

---

**Constructor Summary**

`FbaSolverProcess(Fluxome fluxome)`
  
          Constructs a new FBA solver process.


| **Method Summary** | |
| --- | --- |
| `Fluxome` | `getFluxome()`             Returns the fluxome for which Sv = 0 is solved. |
| `void` | `run(ResultsManager<LinearSystemSolution<java.lang.String>> resultsManager)`             Solves Sv = 0 and adds the result to the provided manager. |
| `void` | `setSolver(LinearSystemSolver<java.lang.String> solver)`             Sets the linear system solver to be used. |

**Methods inherited from class sbrt.kernel.fba.proc.FbaProcess**

`toString`

**Methods inherited from class java.lang.Object**

`clone, equals, finalize, getClass, hashCode, notify, notifyAll, wait, wait, wait`

**Constructor Detail**

### FbaSolverProcess

```
public FbaSolverProcess(Fluxome fluxome)
```

:   Constructs a new FBA solver process.

    **Parameters:**: `fluxome` - the fluxome with for which Sv = 0 is solved.


**Method Detail**

### getFluxome

```
public Fluxome getFluxome()
```

:   Returns the fluxome for which Sv = 0 is solved.

    :   **Specified by:**: `getFluxome` in class `FbaProcess<LinearSystemSolution<java.lang.String>>`
    :   **Returns:**: the fluxome for which Sv = 0 is solved.

---


### setSolver

```
public void setSolver(LinearSystemSolver<java.lang.String> solver)
```

:   Sets the linear system solver to be used.

    :   **Parameters:**: `solver` - the linear system solver to be used.

---


### run

```
public void run(ResultsManager<LinearSystemSolution<java.lang.String>> resultsManager)
```

:   Solves Sv = 0 and adds the result to the provided manager.

    :   **Parameters:**: `resultsManager` - the results manager to which the solution to Sv = 0 will be added.


---


|  |  |  |
| --- | --- | --- |
| **Overview** \| **Package** \| **Class** \| **Use** \| **Tree** \| **Deprecated** \| **Index** \| **Help** | |  |
| **PREV CLASS**   **NEXT CLASS** | **FRAMES**    **NO FRAMES**     **All Classes** |  |
| SUMMARY: NESTED \| FIELD \| CONSTR \| METHOD | DETAIL: FIELD \| CONSTR \| METHOD |  |


---
